# Mutational dynamics of murine angiogenin duplicates

**DOI:** 10.1186/1471-2148-10-310

**Published:** 2010-10-15

**Authors:** Francisco M Codoñer, Silvia Alfonso-Loeches, Mario A Fares

**Affiliations:** 1 Evolutionary Genetics and Bioinformatics Laboratory, Department of Genetics, Smurfit Institute of Genetics, University of Dublin, Trinity College, Dublin, Ireland; 2 IrsiCaixa, Laboratori de Retrovirologia, Hospital Universitari Germans Trias i Pujol, Ctra. Canyet s/n 08916 Badalona Spain; 3 Department of Cellular Pathology, Centro de Investigaciones Principe Felipe, Avda. Autopista del Saler, 16-3 (junto Oceanográfico), 46012 Valencia Spain; 4 Laboratory of Integrative and Systems Biology, Instituto de Biologia Molecular y Celular de Plantas (CSIC- Universidad Politécnica de Valencia (UPV)), Valencia, Spain

## Abstract

**Background:**

Angiogenin (Ang) is a protein involved in angiogenesis by inducing the formation of blood vessels. The biomedical importance of this protein has come from findings linking mutations in Ang to cancer progression and neurodegenerative diseases. These findings highlight the evolutionary constrain on Ang amino acid sequence. However, previous studies comparing human Angiogenin with homologs from other phylogenetically related organisms have led to the conclusion that Ang presents a striking variability. Whether this variability has an adaptive value *per se *remains elusive. Understanding why many functional Ang paralogs have been preserved in mouse and rat and identifying functional divergence mutations at these copies may explain the relationship between mutations and function. In spite of the importance of testing this hypothesis from the evolutionarily and biomedical perspectives, this remains yet unaccomplished. Here we test the main mutational dynamics driving the evolution and function of Ang paralogs in mammals.

**Results:**

We analysed the phylogenetic asymmetries between the different Ang gene copies in mouse and rat in the context of vertebrate Ang phylogeny. This analysis shows strong evidence in support of accelerated evolution in some Ang murine copies (mAng). This acceleration is not due to non-functionalisation because constraints on amino acid replacements remain strong. We identify many of the amino acid sites involved in signal localization and nucleotide binding by Ang to have evolved under diversifying selection. Compensatory effects of many of the mutations at these paralogs and their key structural location in or nearby important functional regions support a possible functional shift (functional divergence) in many Ang copies. Similarities between 3D-structural models for mAng copies suggest that their divergence is mainly functional.

**Conclusions:**

We identify the main evolutionary dynamics shaping the variability of Angiogenin in vertebrates and highlight the plasticity of this protein after gene duplication. Our results suggest functional divergence among mAng paralogs. This puts forward mAng as a good system candidate for testing functional plasticity of such an important protein while stresses caution when using mouse as a model to infer the consequences of mutations in the single Ang copy of humans.

## Background

Angiogenin (Ang) is a 14 kDa protein that belongs to the pancreatic ribonuclease A (RNase) superfamily [1-3], and is involved in angiogenesis by inducing the formation of blood vessels [3,4]. Ang is over-expressed in tumoral cancer cells [5] and inhibition of Ang function through protein-protein interactions blocks the establishment, progression and metastasis in mice [6-11]. Ang may function as a tRNA-specific ribonuclease that binds to actin on the surface of endothelial cells; once bound, angiogenin is translocated to the nucleus, promoting the endothelial invasiveness necessary for blood vessel formation. The biomedical importance of this protein has been recently pinpointed by studies that have associated point mutations in Ang to neuro-degenerative disease as in the case of amyotrophic lateral sclerosis [12-14].

Human Ang (hAng) has been widely studied and has been the first to be isolated from human colon adenocarciroma cells [15]. Crystallization of the hAng protein in 1994 [16] has been instrumental for many molecular and biomedical studies, however little insight has been achieved regarding the structural and functional constraints on Ang mutational dynamics. Despite the important function of Ang, and therefore its expected evolutionary conservation, many research groups found this protein to be evolutionarily variable, probably linked to the divergent function between hAng and angiogenin from other organisms. For example, hAng exhibits a ribonucleolytic activity that is weaker than bovine pancreatic RNase A, around 105 to 106 times less efficient [17-19], probably due to a single amino acid substitution at position 117 of the protein [20]. It cleaves preferentially on the 3' side of pyrimidines and follows a transphosphorylation/hydrolysis mechanism when inducing angiogenesis, differing not only in magnitude but also in the specificity for the bovine pancreatic RNaseA. Whether the angiogenin functional plasticity is correlated with an evolutionary plasticity remains to be tested. To conduct this test it is important to define the set of functional domains and amino acid sites that provide Ang its function and to identify the evolutionary/functional potential of this protein--which refers to the potential of this protein to evolve towards novel functions. Functional and comparative structural analyses have been paramount to unravel key sites for Ang function (See for example, [16,21-23]). Many of these studies have specifically assigned functions to particular amino acid sites within the Ang protein. For example, His13, Lys40 and His114 have been shown to be essential in the catalytic activity of Ang [24-26].

Mouse is the model used to study the implications of mutations at Angiogenin in some human illnesses and studies of murine Angiogenin (mAng) have highlighted a burst of other amino acid sites essential for its activity including: *i*) the B1 binding site comprising Thr44 and Ser118 [16]; *ii*) the poorly conserved B2 binding site, that binds a purine ring on the opposite side of the scissile bond, with Glu108 being key at this functional domain [16,26]; *iii*) the P2 site that facilitates, in conjunction with B2 binding site, the binding of Ang to the nucleus of the cell owing this activity to the amino acids Arg5 and His8 [27]; and *iv*) another putative binding site that has been described to be required for Ang activity, covering the range of residues Asn59 to Asn68, residues Ala108 to Phe110 and residue Asn119. Previous studies pointed to the possible implication of some of these residues (for example residues Glu58 to Lys70) in the binding of Ang to the cell and in causing aggregation rather than purine binding as in the case of RNaseA [28,29]. In addition to these regions, there is a nuclear localization signal that spans amino acids Arg31-Leu35 of mAng [30].

Due to the variable copy numbers for mAng generated by gene duplication, mAng become a questionable model to infer the effect of mutations in hAng, because the functional constraints on amino acid sites in Ang may have changed after gene duplication. This problem is magnified in mice for which six different Ang paralogs genes have been so far described (mAng1 to mAng6), all resulting from tandem duplications of the hAng ortholog (mAng1). Only four out of the six copies (mAng1 to mAng4) have been tested for activity. Among these, mAng1, mAng3 and mAng4 present a ribonuclolytic and angiogenic activity (Nobile *et al*., 1996; Fu *et al*., 1999; Crabtree *et al*., 2007a).

Sites involved in Ang activity have been identified through comparative structural analyses and functional data [31-33]. Moreover, mAng2 has been reported to lack angiogenic activity and has been considered to be a pseudo-gene [31]. To date, no function has been reported for either mAng5 or mAng6. Recent studies have provided evidence for the action of diversifying selection post-dating the duplication events that gave rise to five of the mAng1 paralogs [34-37]. Adaptive evolution has also been found in the duplicated gene of Ang from rats (rAng) [36] and primates [38-40]. Aside from these studies, exhaustive analyses of evolutionary dynamics and structural constraints at this gene in mice remain unperformed.

Here we present an evolutionary study of the duplicated Ang genes in mice to identify amino acid regions that may have played key roles in its functional diversification. We test for the fixation of adaptive amino acid replacements after mAng duplication events to identify shifts on the functional constraints of amino acids after gene duplication and we explore the structural consequences of such shifts. We finally discuss on the putative functional roles of the different Ang proteins in mouse based on our evolutionary analyses.

## Results and Discussion

### Evolutionary history of Ang

**Figure 1:**
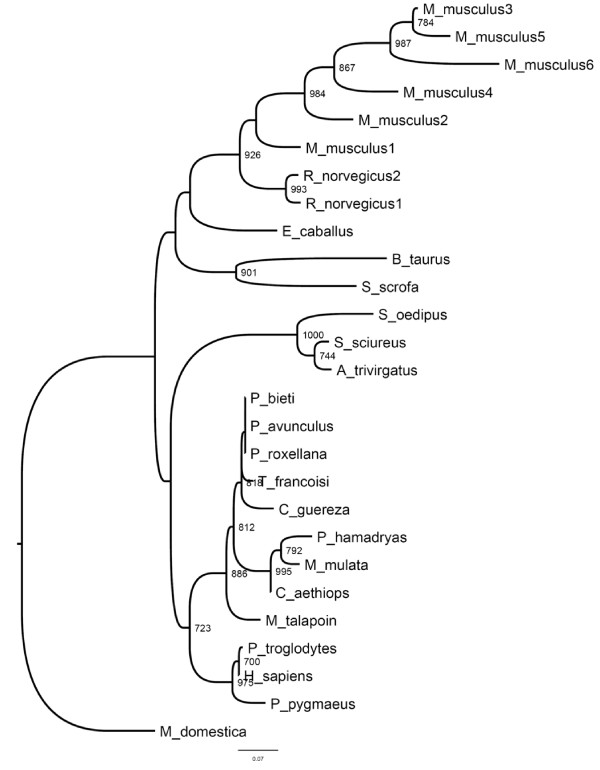
**Phylogenetic reconstruction of the Angiogenin protein**. The topology was inferred by maximum likelihood approach implemented in the PHYML program using the settings for the best evolutionary model, JTT + *Γ *(where the shape parameter *α *of the gamma distribution was estimated to be 0.939). Values at the nodes are bootstrap supports based on 1000 pseudo-replicates. Only bootstrap values greater than 70% are shown.

The aim of this study was to understand the evolutionary dynamics post-dating the different duplication events in mAng. The phylogenetic position of mAng was paramount to infer accurately the evolutionary processes corresponding to each of the multiple duplication events. Maximum-likelihood approach identified *JTT *with a heterogeneous distribution of substitution rates among sites (gamma: *Γ*, with a shape parameter *α *= 0.939) as the best evolutionary model to use in Ang alignments. We tested the support of the phylogenetic position of mAng in two ways. In the first method we inferred the bootstrap support values for each of the node using 1000 alignment pseudo-replicates and inferring the support for each of the nodes. The resulting phylogenetic tree (Figure 1) supported five mouse specific repeated duplication events, while a single duplication could be reliably assigned to the rat lineage (Figure 1). The second approach involved the comparison of the likelihoods for the four alternative phylogenetic positions of mAng. These comparisons were performed using the Hasegawa-Shimodaira (HS) test [41], which is implemented in the program PAML version4. This test pointed to the tree of figure 1 as the most likely hypothesis for the position of mAng1 (for example, this tree minimizes the log-likelihood value of the tree) given our alignments (data not shown). We therefore used this topology in all our downstream evolutionary analyses.

Among the most interesting observations when examining the tree of figure 1 is that repeated *ang *gene duplication events occurred during the radiation of murine and rat clades (Figure 1). These duplication events led to the asymmetric expansion of the Ang proteins in both these lineages with the mouse lineage showing a substantial amount of paralogs (6 paralogs corresponding to five duplication events) as compared to the rat lineage, which showed only two paralogs (Figure 1). Rat paralogs present symmetric branch lengths and hence equal evolutionary rates. Unlike rat, mice paralogs show substantial differences in their evolutionary rates (for example, mAng2, mAng6 and mAng4 present long branches compared to their paralogs) hinting their possible functional divergence after gene duplication. The nature and consequences of this functional divergence are elusive and more analyses are needed to determine whether such divergence led to neo-functionalisation or sub-functinalisation of the paralog copies. Mice present large effective population sizes in comparison to human and hence the probability for neo-functionalisation in mice is greater than in humans. Theoretical and population genetics data predict that in large populations strong constraints act against slightly deleterious mutations, hindering the subsequent fixation of compensatory mutations, and consequently the probability of sub-functionalisation is lower than that of neo-functionalisation [42]. Regardless the final outcome, asymmetry between mice paralogs point to the fixation of burst of mutations by adaptive evolution, which may have driven mAng copies to angiogenin functional diversification. The asymmetry in mice angiogenin paralogs is substantial, but what is the selective value of this asymmetry? and What changes have been essential for mAng functional diversification?

### Diversifying selection has driven the evolution of mAng copies

To identify events of diversifying selection in the murine angiogenin, we applied the maximum likelihood based models implemented in the program CODEML of the PAML package. In addition, we applied the parsimony sliding window approach implemented in the program SWAPSC. This second approach was convenient for several reasons: i) The sliding window approach allows identifying regions of the proteins under diversifying selection, or other non-neutral selective constraints such as accelerated rates of evolution and mutation hotspots; and ii) SWAPSC accounts for nucleotide bias and for non-neutral evolution of synonymous sites.

**Figure 2:**
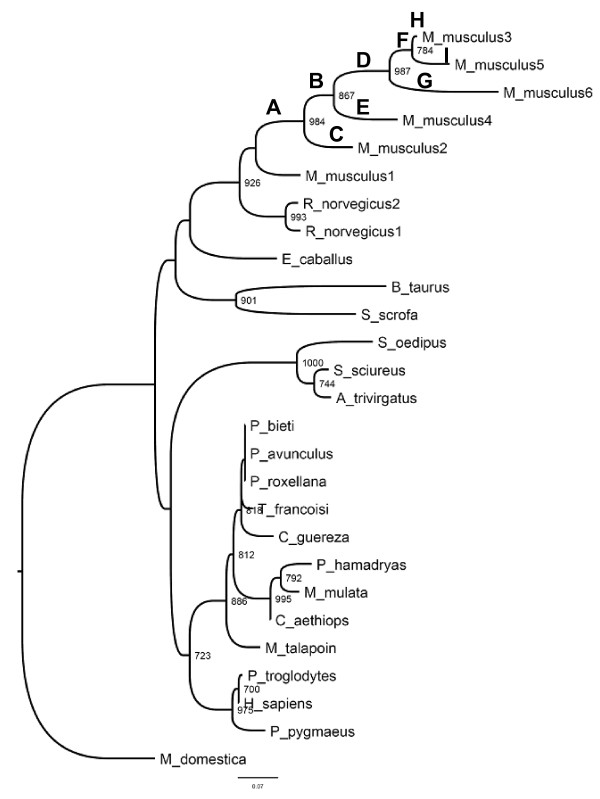
**Selective constraints analysis of pre- and post-duplication lineages**. We used the branch-site model implemented in the program CODEML from the PAML package version 4.0. Branches labeled are those tested in a search for evidence of constraints different from those of the background constraint.

Maximum likelihood based approach supported a heterogeneous distribution of the intensities of selection throughout the phylogeny, as pinpointed by the fact that free-ratio model outperformed significantly the model of Goldman and Yang (Likelihood Ratio Test value *LRT *= χ*^2 ^*= 115.12, *P *= 4.77×10^-7^). Using the intermediate Model B implemented in CODEML, we tested the direct hypothesis of the evolution of each branch before and after duplication events in mice (branches that were tested are indicated in Figure 2). Model B identified several of the branches to have undergone different selection constraints before gene duplication compared to after each of the several gene duplication events. SWAPSC also detected the action of adaptive evolution in specific branches of the tree convergently with maximum likelihood analyses. For example, Branch A (Figure 2), which leads to the ancestor of all the duplication events in the murine lineage, and Branch G, which leads to mAng6, were found to be under positive selection using both approaches. Positive selection at these branches was supported by non-synonymous-to-synonymous rates ratio of ω = 2.3058 and ω = 5.65 in the case of Branch A and ω = 1.731 and ω = 1.16 for Branch G, as computed by maximum likelihood and sliding window analyses, respectively.

Branch F (Figure 2), which leads to the ancestor of mAng3 and mAng5, was detected to be under positive selection using CODEML as the estimated ω = ∞ was greater than one, although synonymous changes were estimated to be 0 and therefore we should be careful in interpreting this result. In Branch A we found several sites under PS, including amino acids spanning the region between Met30 and Gly34. Importantly, this region includes the sites responsible for the nuclear localization of the protein [43]. At Branch G several sites were found to be under positive selection using both approaches, including residues Ser52 to Gly62. Although the functional importance of some of these amino acids is as yet unknown, some of the residues detected to have undergone positive selection have been reported to be close or directly involved in the nuclear localization region of mAng (for example amino acids included in the region Arg31 to Leu35) [43]. This could be an indication that functional divergence of mAng6 was due to positively selected amino acid replacements at these sites.

Inspecting the rest of the branches leading to the different duplicates we found that branch H, which leads to mAng3 (Figure 2), seems to have been evolving under negative selection, with ω = 0.1912 and ω = 0.1329 for maximum likelihood and sliding window, respectively. In contrast some branches such as branch D, which leads to the ancestor of mAng3, mAng5 and mAng6, seems to have been evolving neutrally (ω = 0.951). Similar results were obtained for branch E (ω = 1.015), that corresponds to the mAng4 lineage and branch I (ω = 0.964), that leads to mAng5. This neutral evolution points to the possible non-functionalization of these copies, but as mAng4 and mAng3 have been shown to be functional, we expected mAng5 to be functional as well; we show further analysis that point to this hypothesis as the most plausible one.

Interesting was the case of branch C, mAng2 (Figure 2), because it seems to have been evolving at the same rate (ω = 0.618) as the rest of the species included in the phylogenetic analysis (ω = 0.556). It has been described that mAng2 is a possible pseudo-gene, however we have not detected any evidence for relaxed constraints in this lineage in comparison with other lineages of the mammalian tree that could suggest neutral fixation of amino acid replacing nucleotide substitutions (for example ω = 0.556 < < 1). Conversely, mAng2 seem to have undergone moderate purifying selection.

Gene's expression is one of the main factors affecting evolutionary rates, with expressed genes being those highly conserved. To account for this when comparing non-synonymous-to-synonymous rates ratios among mAng gene copies we investigated the expression of each of the copies using codon adaptation index (CAI) as a proxy to gene expression. CAI was calculated using the webpage http://www.cbib.u-bordeaux2.fr/pise/cai.html. The values of non-synonymous-to-synonymous nucleotide substitutions are not due to different expression levels of the gene copies because, on average, the different mAng copies presented similar expression levels (CAI was estimated to be 0.245, 0.235, 0.232, 0.247, 0.243 and 0.237 for mAng copies 1 to 6, respectively). These gene copies also presented similar expression levels to that of hAng (CAI = 0.262). Difference in evolutionary rates therefore was not due to differences in expression levels among duplicates.

The fact that these copies remain in the proteome of mouse argues against previous studies suggesting non-functionalisation [31]. In addition, all post-duplication lineages presented similar intensities of selection except the pairs of post-duplication lineages F-G (leading to mAng6 and ancestor of mAng5-mAng3 respectively) and H-I that lead to mAng5 and mAng3, respectively (Figure 2). The elevated ω values are more consistent with shifts in the evolutionary rates after gene duplication and with the possible functional divergence of the resulting paralogous copies. In the first pair (F-G lineages), both post-duplication lineages underwent adaptive evolution (for example ω > 1) indicating the possible functional divergence type II (as defined in [44]). Functional divergence type II involves a change of the ancestral amino acid at a particular amino acid site of the protein after gene duplication. This replacement involves the fixation of two different residues in the post-duplication lineages and their high conservation after the speciation of each of the copies due to their different but equally important functional role in each of the paralogs. Conversely, both post-duplication lineages in the second pair (H-I) evolved under purifying selection, although mAng5 presented significantly accelerated rates of evolution compared to mAng3, indicating possible functional divergence type I (as defined by [45]). Unlike functional divergence type II, type I involves the fixation of a function conferring residue mutation in one of the paralogs where it becomes highly constrained, while this amino acid sites evolves neutrally in the other where amino acid replacements occur with no functional consequences.

### Co-evolution between residues proximal to functional regions in Ang

Relaxed selection is a common phenomenon after gene duplication and it can take place in one or both copies of the gene because of gene redundancy [46,47]. One of the gene copies therefore may accumulate deleterious mutations while the other copy can remain under strong purifying selection to preserve the ancestral function. The most expected fate for one of the gene copies is non-functionalization followed by its disintegration within few million years of evolution depending on the effective population sizes of the organism [48]. The two copies of a gene can persist in the genome either if the combined function of both paralogs performs the ancestral function (sub-functionalization) or if one copy reproduces the ancestral function while the other diverges towards other functions (neo-functionalisation). Survival of a pseudo-gene in the genome for long evolutionary periods is very unlikely, and therefore copies that remain are likely to be functional. However, evolution of gene copies after duplication can be very complex and up to twelve models have been recently proposed to account for all possible evolutionary scenarios [49]. Based on this assumption, we examined whether the *mAng *gene copies that were kept in the genome were followed by functional divergence after duplication. Functional divergence is likely to happen in two ways: i) classic functional divergence involves the accumulation of functionally innovative advantageous mutations in one of the gene copies [44,45]; or alternatively ii) after gene duplication functionally innovative but structurally destabilizing mutations may have become fixed once they have been compensated for by other mutations (compensatory co-evolution): in a normal physiological background the effect of both two mutations is neutral but the phenotypic advantage of the destabilizing mutation may be expressed under novel environmental conditions.

Applying the method of Gu [45] we could not identify classical functional divergence in any of the considered clusters. To identify the second type of functionally divergent mutations we first performed analysis of co-evolution (see Material and methods for details). The co-evolution method identified several pairs of amino acids showing correlated changes. Groups of coevolution --with each group including only amino acids that present correlated evolution with each and all the members of that group (Table 1)-- highlighted several amino acid sites to be correlated in their evolutionary patterns (Table 1). Most of the sites are close (for example within 4Å) to essential amino acids of the active site (His13, Lys40 and His114), or to the binding sites, or to the domain responsible for the nuclear translocation (Arg31-Leu35). These proximities support the possible compensatory relationship between such amino acid sites because their proximity to important functional regions makes it likely that mutations at these sites can have deleterious effects. The next question we asked was whether these constraints have undergone substantial changes after gene duplication. To answer this question and the hypothesis of compensatory effects we analysed the distribution of co-evolving pairs of amino acid sites in the protein structure and tested their proximities.

**Table 1:** Residues in the Ang protein involved in intramolecular coevolution

Groups of coevolution	Amino acids
G1	S4, K60
G2	S4, N102
G3	I29, R66
G4	D41, N49
G5	D41, N59
G6	D41, N61
G7	D41, N63
G8	D41, E67
G9	D41, K82
G10	L69,K73,Q93,A98,N102
G11	L69,T80
G12	K73,H84,Q93,T97,A98
G13	K73,Q93,A98,N102,G110
G14	K82,P88
G15	K82,Q93,T97,A98,N102
G16	H84,W89,Q93,T97
G17	H84,A98,G110
G18	H84,W89,121
G19	W89,G110

Amino acid position and residue (in one-letter code taking as a reference sequence the *H. sapiens *sequence from the 1ANG pdb file) of the detected amino acids is included.

### Detection of compensatory mutations

**Figure 3:**
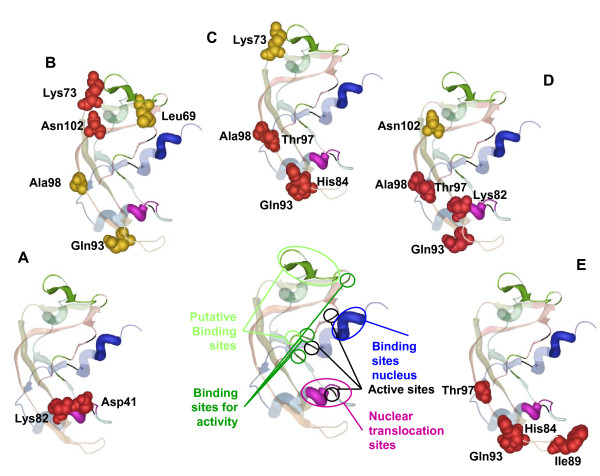
**Analysis of compensatory mutations**. Ang protein where all the described functional domains are highlighted in different colors is shown in the middle of the figure, black colored amino acids are sites involved in the active site, blue colored are the ones involved in binding to the nucleus while purple sites are the ones involve in nuclear translocation of the protein. Dark green color shows the binding site while light green highlight the putative binding site. Co-varying sites are shown in a space fill format, and only those sites detected as compensatory are colored in red. (A), (B), (C), (D) and (E) are co-varying sites from group G9, G10, G12, G15 and G16 respectively.

In order to understand the relationships between co-evolving amino acids in Ang, we plotted these in the crystal structure of hAng and asked whether pairs of co-evolving residues presented evidence of interaction with one another. In spite of the fact that mAng1 and mAng4 have been isolated and crystallized, we used hAng as a reference structure due to the medical relevance of this protein for humans and because it was identical to mAng. All other mouse Ang structure copies have been synthetically modified from the mAng1 in previous studies. The procedure utilized to answer this question consisted in determining whether the pair of co-evolving residues was located within 4Å from each other, being indicative of their possible functional or structural interactions. Alternatively, for those distantly located amino acids (presenting a distance from one another greater than 4Å), we asked whether they were contacting common amino acids that showed highly conserved evolutionary pattern (see material and methods for details). Many of the co-evolving amino acids presented distances greater than 4Å (Figure 3). Importantly, most of these amino acids were proximal to residues that showed a significantly conserved evolutionary pattern compared to the rest of the alignment (pairwise Poisson distances in the lower 99% tail of the distances distribution, see methods for details). This method was used previously with significant success to identify compensatory relationships between mutations [50]. The highly conserved sites identified nearby co-evolving residues are close to the sites responsible for the ribonuclease activity and to those involved in the translocation of the protein to the nucleus.

After examining the different co-evolution groups we found that groups G9, G10, G12, G15 and G16 (Table 1) presented pairs of amino acids with strong evidence for their compensatory effects--that is to say they fall within 4 Å of each other in the protein structure and are therefore likely to present interacting effects. The pair of amino acid sites Asp41-Lys82 (Figure 3A), classified within co-evolution group G9 (Table 2), presented evidence of compensatory effects. Asp41 and Lys82 are involved in dimerization of angiogenine and are closely located to amino acid regions that interact with inhibitors and the catalytic centre (Table 2). Another example of possible compensatory interaction is that presented by the pair of amino acid sites Lys73-Asn102 (Figure 3B) that are classified within group G10 (Table 2). Importantly, this pair of amino acids is located structurally close to the putative binding site (Figure 3). His84-Gln93 and His84-Thr97 where detected as compensatory and they are in two co-evolutionary groups, G12 (Figure 3C and Table 2) and G16 (Figure 3E and Table 2). These sites are close to catalytic sites and are both involved in binding the inhibitor of angiogenin. The pair His84-Ile89 was also found as a coadaptation pair in G16 (Figure 3E and Table 2). Another pair Thr97-Ala98, which has been consistently detected to co-evolve in many of the groups, also presented evidence for a compensatory relationship in G12 (Figure 3C and Table 2) and G15 (Figure 3D and Table 2). Thr97 and Ala98 are involved in angiogenin dimerization (Table 2). In G15 (Figure 3D and Table 2) we also found other compensatory mutations Lys82-Gln93 and Lys82-Thr97. All compensatory mutations have been highlighted in red in Figure 3. It is interesting to notice that, although many of the sites detected to be under adaptive evolution fall within the same domains of those co-evolving (for example, sites 30 to 35 which are within the nuclear localization signal peptide), there was no match between these two sets of sites. One possible reason may be purely methodological because both selection and functional divergence analyses have been performed in a qualitatively different manner. In selection analyses we focused the detection of adaptation on particular lineages of the tree. Conversely, in co-evolution the entire tree was used which makes it more difficult to identify selection at co-evolving amino acid sites: pairs of amino acids that changed in a correlated way in few lineages may have undergone strong purifying selection in most of the remaining lineages of the tree. This would imply that on average these sites would be under strong negative selection most of the time alternating with punctual episodic adaptive evolution, which would be unlikely to be detected by actual selection methods. We consider therefore both the selection methods and co-evolutionary analyses to be complementary approaches to identify adaptive evolutionary events.

**Table 2:** Identification of functionally important residues using co-evolutionary analyses

Pairs	Coevolution Group	Conserved Sites (4Å close)	Active Site	Binding Site	Putative Binding Site	Nuclear Implications
D41-K82	G9	H84, Q93, R95	K40, D41, I42, C92	T80, C81, K82, R121	-	-
K73-N102	G10, G13	S74, S75, N102	-	R101	I56, N63, R70, I71, S72, K73, R101	
K82-Q93	G15	D23, H84, Q93, R95, A96	K40, D41, C92	C81, K82	-	C26, E27
K82-T97	G15	D23, H84, R95, A96, T97, R122	-	T44, T79, C81, K82, F120	-	C26
H84-Q93	G12, G16	D23, H84, G86, P91, Q93, R95, A96	C39, K40, D41, S87, C92	C81, K82	-	E27, C39
H84-T97	G12, G16	D23, H84, R95, A96, T97	-	C81, K82	-	-
W89-Q93	G16	T36, G86, W89, P90, P91, Q93	C39, K40, S87, C92	-	-	-
T97-A98	G12, G15	R21, D22, H47, Q77, R95, A96, T97, A98, G99, F100	-	V78, T79, T80, C81	V78	C26
D41-K82	G9	H84, Q93, R95	K40, D41, I42, C92	T80, C81, K82, R121	-	-
K73-N102	G10, G13	S74, S75, N102	-	R101	I56, N63, R70, I71, S72, K73, R101	
K82-Q93	G15	D23, H84, Q93, R95, A96	K40, D41, C92	C81, K82	-	C26, E27
K82-T97	G15	D23, H84, R95, A96, T97, R122	-	T44, T79, C81, K82, F120	-	C26
H84-Q93	G12, G16	D23, H84, G86, P91, Q93, R95, A96	C39, K40, D41, S87, C92	C81, K82	-	E27, C39
H84-T97	G12, G16	D23, H84, R95, A96, T97	-	C81, K82	-	-
W89-Q93	G16	T36, G86, W89, P90, P91, Q93	C39, K40, S87, C92	-	-	-
T97-A98	G12, G15	R21, D22, H47, Q77, R95, A96, T97, A98, G99, F100	-	V78, T79, T80, C81	V78	C26

We identify highly conserved amino acid sites (labeled in one-letter code and using as reference the *H. Sapiens *sequence) that are structurally close to or within functionally important regions of Ang protein and that are close (within 4Å) to coevolving pairs with potential compensatory relationships. In **bold **we highlight those co-evolving amino acid positions reported to be involved in dimmer formation of hAng, in *Italic *we remark positions implicated in the interaction with angiogenin-inhibitor in Human. We finally underscore those positions identified to be important as catalytic sites in hAng, as described in NCBI-IBIS database [78].

### Structural bases of amino acid variability in mAng

**Figure 4:**
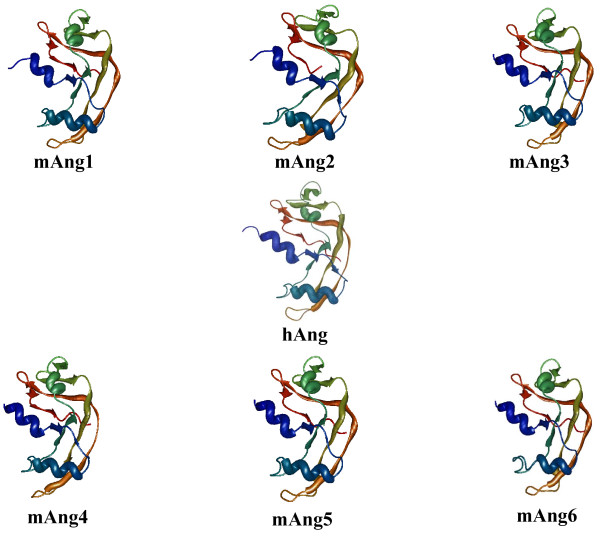
**Three-dimensional modeling of the duplicated Angiogenin proteins**. These structures were modeled by homology and the details are gathered in the Results section. hAng, mAng1 and mAng4 structures belong to the 1ANG, 2BWL and 2J4T protein databank files, respectively.

To understand the structural basis of amino acid variability among the mAng copies, we modelled three-dimensional structures for the different mAng copies that lack such structure (mAng2, mAng3, mAng5 and mAng6) by homology using the program 3D-JIGSAW [51,52]. Several Ang structures are available in the databases, including three belonging to mice (with accession numbers 2J4T, 2BWL and 2BWK) and many other belonging to synthetic versions of humans or resulting from different approaches of crystallization. Two of the mAng structures belong to mAng1 (2BWL and 2BWK) but have been crystallized using different protocols, while a third structure (2J4T) belongs to mAng4. We however adopted the hAng (1ANG) as a reference to number all sites because the remaining human structures are highly similar in sequence and structure to this one. To identify differences among the six copies of mouse Angiogenin proteins' structures we used the program CCOMP, which measures the distance between the mass centres of amino acids of two structures by the Root Mean Square Deviation [53]. Although we observed no important differences in the structure among the Ang copies (for example, all six copies present less than 2Å root mean square deviations from the first copy; Table 3), our results point to the possible blockage of the active site in mAng2, which may explain the lack of mAng1-like activity (Figure 4). The similarity at the structural level but the difference regarding the functional site supports the hypothesis of functional shift after the duplication, which may have given rise to mAng2 and its departure from an Angiogenic activity. This result also argues against the non-functionalization of any of the mouse Ang copies. Unlike mAng2, mAng4 has been previously reported to be active [13,32], although we observed an important difference in the structural configuration of the active centre when compared to mAng1--that is to say, the active centre of one copy was structurally displaced and presented slight structural differences when compared to the other copy (Figure 4).

**Table 3:** Root Mean Square Deviation (RMSD) between the modeled structures for murine ANG protein paralogs

Comparison	RMSD
mAng1 vs mAng2	1.078
mAng1 vs mAng3	0.772
mAng1 vs mAng4	0.814
mAng1 vs mAng5	0.744
mAng1 vs mAng6	1.038
hAng vs mAng1	1.306

Similarly to mAng2, mAng5 shows a structural departure from mAng1 in the active centre with evidence of activity blockage (Figure 4). This supports the previously observed poor angiogenic activity and the suggested involvement of mAng in other processes [31].

Finally, our selective constraints analyses show evidence of adaptive evolution in mAng6. The fact that our structural modelling detects a structurally different active centre in this protein compared to other protein copies where we detect adaptive evolution suggests functional divergence after the split between mAng6 and the remaining Ang copies. Furthermore, intra-molecular co-evolutionary analyses show evidence of compensatory mutations events located nearby important functional regions of the Ang protein. For example, Pro18 and Thr36, frequently identified as coevolving with many other residues in the structure, are probably responsible of the operability of the active site as its location suggests its role in maintaining the proper orientation of sites His13 and Thr44 that belong to the active site. The rest of the pairs of coevolution are mostly surrounding the active site--which comprises amino acids His13, Thr44 and His114. Others are either included within or surrounding the nuclear peptide signal (Arg31-Leu35). These results indicate therefore that, in general, amino acid sites close or included in important protein domains may have coevolved to maintain the structural features necessary for the proper functional activity of Angiogenin. We have also detected two groups of compensatory mutations in mAng after duplication. The importance of these sites is further enhanced by their location in or nearby amino acids Arg31 to Leu35 that have been described to be involved in the nuclear localization of the protein in mouse [30].

## Conclusions

Even though all the duplicates in mouse are different from mAng1, and that nothing has been described for mAng5 and mAng6, there is no reason to think that these copies are non-functional. Remarkably it has been reported the non-nuclear localization of mAng6. The identification of positive selection in amino acids involved in the nuclear localization of the protein may be related to a functional shift of this angiogenin mouse copy. Moreover, mAng2 seems to be functional in contrast to previous reports.

In conclusion, our analyses yield novel results that shed light on the mutational dynamics of Angiogenin when exploring diversifying natural selection and functional divergence. We present the structural and functional interpretations for these evolutionary dynamics and provide a list of amino acid sites that are likely to have a functional impact on the mAng copies suitable for further experimental testing. We also reject the previously supported non-functionalization of duplicated mAng based on evolutionary and structural analyses and we invite researchers for a rather more detailed investigation of the roles and redundancies of duplicated Angiogenin genes.

## Methods

### DNA and protein sequences

Table 4 contains a list of the accession numbers for the protein and DNA sequences used in this study. Sequences were all downloaded from the NCBI database http://www.ncbi.nlm.nih.gov after a BLAST search (Altschul *et al.*, 1997) using the human angiogenine (hAng) as the query sequence. Protein sequence alignments were obtained using MUSCLE v3.7, with the default parameters [54,55]. We then aligned protein-coding nucleotide sequences by concatenating triplets of nucleotides according to the amino acid alignment (multiple sequence alignment for nucleotides and proteins are depicted in Additional file 1, Figure S1 and Additional file 2, Figure S2, respectively).

**Table 4:** Accession numbers for the DNA and protein sequences of the Angiogenin protein used in the analysis

Species	Protein	DNA
Mus musculus1	NP_031473	NM_007447.2
Mus musculus2	NP_031475	NM_007449.2
Mus musculus3	AAC05794	U72672
Mus musculus4	NP_808212	NM_177544
Mus musculus5	AAV87188	AY665820
Mus musculus6	AAV87189	AY665821
Rattus norvegicus1	NP_001006993	NM_001006992.1
Rattus norvegicus2	NP_001012359	NM_001012359.1
Homo sapiens	NP_001136	NM_001145.2
Trachypithecus francoisi	AAO41336	AY221129
Pygathrix avunculus	AAO41339	AY221132
Pygathrix bieti	AAO41338	AY221131
Pygathrix roxellana	AAO41337	AY221130
Pongo pygmaeus	AAL61645	AF441663.1
Chlorocebus aethiops	AAL61646	AF441664
Sus scrofa	NP_001038038	NM_001044573
Miopithecus talapoin	AAL61647	AF441665
Pan troglodytes	NP_001009159	NM_001009159
Macaca mulatta	AAL61649	AF441667
Equs caballus	NP_001075368	NM_001081899
Saguinus oedipus	AAL61650	AF441668
Bos Taurus	NP_001071612	NM_001078144
Saimiri sciureus	AAL61652	AF441670
Aotus trivirgatus	AAL61651	AF441669
Papio hamadryas	AAL61648	AF441666
Colobus guereza	AAO41335	AY221128
Monodelphis domestica	XP_001379328	XM_001379291

### Phylogenetic reconstruction

A molecular evolutionary model was first fitted to the protein alignment using PROTTEST 1.0.6 [56]. Phylogenetic trees for proteins were obtained using the PHYML [57] with the best evolutionary model found in PROTTEST. Confidence of the nodes of the tree was tested by first building 1000 bootstrap pseudo-replicate alignments using the BOOTSTRAP program implemented in the PHYLIP package v3.67 (J. Felsenstein, U. Washington, freely available at http://evolution.genetics.washington.edu/phylip/getme.html). We then used PHYML to obtain 1000 trees from these replicates. The consensus tree was obtained using the majority rule approach implemented in CONSENSE program in the PHYLIP package.

### Identification of selective constraints

To identify the main functional diversifying events in Ang during the evolutionary radiation of mammals we analyzed the change in the dynamics of synonymous (d_S_) and of non-synonymous (d_N_) nucleotide replacements. In our study we assumed that d_S _accumulates neutrally on average since they produce no amino acid replacements and are therefore not seen by selection. Taking into account this assumption, we estimated the intensity of selection by obtaining the ratio between d_N _and d_S _(*ω *= d_N_/d_S_). This ratio has been regarded as the most stringent way to identify selection, with *ω *= 1, *ω *< 1 and *ω *> 1, indicating neutral evolution, purifying selection and diversifying selection, respectively [58-60]. However, caution is required when measuring selection using this approach because the stability of RNA molecule secondary structure as well as translational selection may impose constraints on synonymous sites leading to lower d_S _values and consequently to inflated ω estimates [61-64].

To ameliorate the effects of these limitations, we tested for the presence of diversifying selection following two main ways. First we used maximum-likelihood models to identify selective constraints as implemented in the program CODEML of the PAML package v4.0 [65]. Using this approach, we compared a model assuming homogenous distribution of selective constraints along the protein and the phylogeny (model M0: one ω value for the entire tree and alignment) to a model assuming an independent ω for each lineage of the tree [66]. These two nested models (the more complex model includes parameters of the simple model) were compared by the likelihood ratio test (LRT) [67], with twice the difference between the log-likelihood values of the two models being compared to a χ^2 ^distribution with as many degrees of freedom as number of branches in the tree -1. Second we used a parsimony-based approach robust to deviations from the assumption of neutrality of synonymous substitutions. This parsimony approach was based on the sliding window procedure previously published [68] and is implemented in the program SWAPSC version 1.0 [69]. This program uses a statistically optimized window size to detect selective constraints in specific codon regions of the given alignment at a particular branch of the phylogenetic tree that show the evolutionary history of the sequences under study [68].

Briefly, SWAPSC estimates the expected distribution of d_S _and d_N _by Li's method [70] from simulated alignments and assuming a Poisson distribution of substitutions. A statistically optimum windows size is then estimated that makes the detection of adaptive evolution independent of the windows size. The empirical values of d_S _and d_N _obtained by using the optimal window size are contrasted with the expected distributions, and several hypotheses regarding the selective constraints acting on codon regions are tested. We obtained the simulated alignments needed for the analysis with the EVOLVER program implemented in the PAML package version 4.0, with the parameters estimated from the true sequence alignment after running the most appropriated codon based model in PAML. Finally, we considered only regions and branches detected under adaptive evolution by those approaches as the true positive results.

### Detection of intra-molecular co-evolution

To test for intra-molecular coevolution, we used a recently developed parametric model [71] implemented in the program CAPS v1 [72]. The sensitivity of CAPS to identify coevolution between pairs of amino acid sites that are functionally linked has been shown to outperform other methods based on mutual information content or on other models of coevolution [71]. We considered therefore the method to be appropriate for an accurate detection of co-evolution. This method has been applied in numerous case studies similar to the one here conducted [50,71,73,74].

Briefly, CAPS compares the correlated variance of the evolutionary rates at 2 amino acid sites in a protein alignment, corrected by the time since the divergence of the 2 sequences they belong to. The algorithm estimates the synonymous nucleotide pairwise sequence divergence as a proxy for their divergence time. This method compares the amino acid transition probability scores between 2 sequences at 2 particular sites, using the blocks substitution matrix [75]. The significance of the CAPS correlation values was assessed by randomly pairing sites of the alignment and building a distribution of correlation coefficients for 1,000,000 randomly paired sites against which we compared real correlation values. To correct for multiple tests and data non-independence CAPS performs a step-down permutation procedure [76] and corrects the probabilities for the correlation coefficients of co-evolving pairs of sites accordingly [72].

For co-evolution analyses we used the protein-coding sequence of Ang and minimized type I error using a confidence value of 0.01. The structural PDB file for hANG (1ang, [16]) was used to identify the co-evolving amino acid positions in the structure (for example, all the amino acid positions in this study refer to their location in the hAng three-dimensional structure).

Molecular co-evolution between amino acids can be the result of their structural, functional, interaction, phylogenetic, or stochastic link [77]. Disentangling the different types of coevolution is a difficult task, although a phylogenetic approach has been suggested as a feasible way to remove amino acids covariation due to stochastic noise [71]. Distinguishing between structural, functional, and interaction co-evolution requires biological information in addition to the mathematical adjustments made by the method. Accordingly, we used correlated variation in the physico-chemical properties of the amino acids as a further filter to our co-evolutionary analyses.

### Identifying Compensatory Mutational Dynamics in Angiogenin

Each one of the amino acid sites identified as coevolving was plotted in the crystal structure of the protein and the Euclidean distance between them was calculated. We calculated this distance as the average distance between the atoms of the amino acid sites as follows:

$$d=\frac{1}{NK}\sum_{i=1}^{N}\sum_{j=1}^{K}\sqrt{{\left(X_{i}-X_{j}\right)}^{2}+{\left(Y_{i}-Y_{j}\right)}^{2}+{\left(Z_{i}-Z_{j}\right)}^{2}}$$

Here *N *is the number of atoms in amino acid *i *while *K *is that number in amino acid *j*. X, Y and Z represent the three-dimensional coordinates of the atoms corresponding to each of the amino acids. We considered two amino acids to contact each other when the distance between their closest atoms was equal or less than 4Å.

Two mutations were considered to have compensated each other if, in addition to presenting the same phylogenetic pattern (be coevolving), they were located within 4Å from each other in the protein crystal structure. Also, two amino acid sites can compensate each other indirectly. For example, if site "A" and site "B" are at more than 8Å distance but are surrounding (within 4Å) an important functional site "C", then changes at site "A" may affect site "C" which has to be compensated by changes at site "B". We also considered these cases to be in support of a compensatory relationship between sites "A" and "B". However, caution must be taken in making such assumptions because close amino acids, even though are likely to influence one another, may not have a compensatory relationship. Conversely, amino acid sites distantly located in the protein structure may have indirect compensatory effects upon one another. The other limitation of this approach is that proteins can undergo dramatic conformational changes during their interactions with other proteins, which is not reflected in the crystal (static) structure of proteins. Under these circumstances, amino acids that are distantly located may interact and hence influence one another. Nonetheless, we adopted the very conservative view that amino acids interacting in our crystal structures are true interactors at the particular conditions under which the protein was crystallized. To identify compensatory relationships under our assumptions between amino acids at distances greater than 4Å, we searched for sites contacting both covarying amino acid sites in the structure showing very low divergence levels in comparison with the rest of the molecule. We measured divergence levels per site by estimating the Poisson amino acid distances for each amino acid site in the multiple sequence alignments. The level of divergence was compared to the distribution of divergence levels built using a pseudo-random sample of 1,000,000 amino acid site columns sampled with replacement from the alignment (one site could be sampled more than once).

### Three-dimensional analysis in Angiogenin

3D-JIGSAW program [51,52] was used to model the 3D structure of the different duplicates. To identify differences among the six copies of mouse Angiogenin proteins' structures we used the program CCOMP [53], that measures the Mean Root Square Deviation between the different structures.

## Authors' contributions

MAF and FMC conceived the idea in collaboration with SAL. FMC and MAF did the analyses. FMC drafted the manuscript and MAF wrote the final version of the manuscript. All authors read and approved the final version of the manuscript.

## Supplementary Material

Additional file 1 **Figure S1**. Multiple sequence alignment for nucleotides and proteins Click here for file

Additional file 2 **Figure S2**. Multiple sequence alignment for nucleotides and proteins Click here for file
