# Supplementary material for: Mutational dynamics of murine angiogenin duplicates
Source: BMC Evol Biol. 2010 Oct 15;10:310. doi: 10.1186/1471-2148-10-310 (PMC2964713; doi:10.1186/1471-2148-10-310)
Supplement: Additional file 1 — Figure S1. Multiple sequence alignment for nucleotides and proteins [file 1471-2148-10-310-S1.pdf]

```
                    10        20        30        40        50        60        70        80
           ....|....|....|....|....|....|....|....|....|....|....|....|....|....|....|....|....
H_sapiens      ATGGTGATG---------GGCCTGGGCGTTTTGTTGTTGGTCTTCGTGCTGGGTCTGGGTCTGACCCCACCGACCCTG---GCT
M_musculus1    ATGGCGATA---------AGCCCAGGCCCGTTGTTCTTGATCTTCGTGCTGGGTCTGGTTGTGATCCCTCCCACTCTG---GCT
M_musculus2    ATGGCGATG---------AGCCCAGGTCCTTTGTTCTTGGTCTTCCTGTTGGGTCTGGTTGTGATCCCTCCCACTCTG---TCT
M_musculus3    ATGGTGATG---------AGCCCAGGTTCTTTGTTGTTGGTCTTTTTGCTGAGTCTGGATGTGATCCCTCCCACTCTG---GCT
M_musculus4    ATGACAATG---------AGCCCATGTCCTTTGTTGTTGGTCTTCGTGCTGGGTCTGGTTGTGATTCCTCCAACTCTG---GCT
M_musculus5    ATGGTGATC---------AGCCCAGGTTCTTTGTTGTTGGTCTTTTTGCTGAGTCTGGATGTGATCCCTCCCACTCTG---GCT
M_musculus6    ATGGTGATG---------AGCCCAGGTTCCTTGATGTTGGTCTTTGTGCTGAGTCTGGTTGTGATCTCTCCCACTCTG---GCT
R_norvegicus1  ATGGAGATG---------AGCCTGCGTCCTCTGTTGTCGGTTTTTGTGCTGGGTCTGGTTTCGACCCCTTCAACTCTG---GCT
R_norvegicus2  ATGGAGATG---------AGCCTGCGCCCTCTGTTGTTGGTTTTTGTGCTGAGTCTGGTTGCGACCCCTTCAACTCTG---GCT
T_francoisi    ATGGTGATG---------GGCCTGGGCCTTTTCTTGTTGGTCTTCATGCTGGGTCTGGGTCTGACCCCACCCACCCTG---GCT
P_avunculus    ATGGTGATG---------GGCCTGGGCCTTTTCTTGTTGGTCTTCATGCTGGGTCTGGGTCTGACCCCACCCACCCTG---GCT
P_bieti        ATGGTGATG---------GGCCTGGGCCTTTTCTTGTTGGTCTTCATGCTGGGTCTGGGTCTGACCCCACCCACCCTG---GCT
P_roxellana    ATGGTGATG---------GGCCTGGGCCTTTTCTTGTTGGTCTTCATGCTGGGTCTGGGTCTGACCCCACCCACCCTG---GCT
P_pygmaeus     ATGGTGATG---------GGCCTGGGCGTTTTGTTGTTGGTCTTCATGCTGGGTCTGGGTCTGACCCCACCAACCCTG---GCT
C_aethiops     ATGGTGATG---------GGCCTGGGCCTTTTCTTGTTGGTCTTCATGCTGGGTCTGGGTCTGACCCCACCCACCCTG---GCT
S_scrofa       ATGGTGATA---------TTGCTGGGCCCCCTGCTGTTGGTCTTCATGCTGGGTCTGGGTCTGGCCCCGCTGAGCCTG---GCT
M_talapoin     ATGGTGATG---------GGCCTGGGCCTTTTCTTGTTGGTCTTCATGCTGGGTCTGGGTCTGACCTCACCCACTCTG---GCT
P_troglodytes  ATGGTGATG---------GGCCTGGGCGTTTTGTTGTTGGTCTTCGTGCTGGGTCTGGGTCTGACCCCACCGACCCTG---GCT
M_mulata       ATGGTGATG---------GGCCTGGGCCTTTTCTTGTTGGTCTTCATGCTGGGTCTGGGTCTGACCCCACCCACCCTG---GCT
E_caballus     ATGGCGATG---------AGCCTGTGCCCCCTGTTGTTGGTCTTCGTGCTGGGTCTGGGTCTGACCCCACCATCCCTG---GCT
S_oedipus      ATGGTGATG---------GGCCTGCACCTTTTGTTTTTGGTCTTCATTCTGGGTCTGGGTCTGACCCCACCAACTCTG---GCT
B_taurus       ATGGTCATG---------GTCCTGAGCCCCCTGTTTTTGGTCTTCATACTGGGTCTGGGTCTGACCCCAGTGGCCCCG---GCT
S_sciureus     ATGGTGATG---------GGCCCGCACCTTTTGTTGTTGGTCTTCATTCTGGGTCTGGGTCTGACCCCACCGACTCTG---GCT
A_trivirgatus  ATGGTGATG---------GGCCTGCATCTTTTGTTGTTGGTCTTCATTCTGGGTCTGGGTCTGACCCCACCGACTCTG---GCG
P_hamadryas    ATGGTGATG---------GGCCTGGGCCTTTTCTTGTTGGTCTTCATGCTGGGTCTGGGTCTGACCCTACCCACCCTG---GCT
C_guereza      ATGGTGATG---------GGCCTGGGCCTTTTCTTGTTGGTTTTCCTGCTGGGTCTGGGTCTGACCCCACCCACCCTG---GCT
M_domestica    ATGGTGATGTTTCTTAAGGCCATGGACCCTTTCTTGCTGCTCTTCATCCTGGGGCTATGGCTGGTTCCACCTTCCTTGACCACA

                    110       120       130       140       150       160       170       180
           ....|....|....|....|....|....|....|....|....|....|....|....|....|....|....|....|....
H_sapiens      ACACACACTTCCTGACCCAGCACTATGATGCCAAACCACAGGGCCGGGATGACAGATACTGTGAAAGCATCATGAGGAGACGGG
M_musculus1    ACACAAAATTCCTGACTCAGCACCATGACGCCAAGCCAAAGGGCCGGGACGACAGATACTGTGAACGTATGATGAAGAGAAGAA
M_musculus2    ACACAAAATTCCTGACTCAGCACTATGATGCCAAGCCAAAAGGCCGGGACGACAGATACTGCGAAAGTATGATGGTGAAAAGAA
M_musculus3    ACATAAAATTCCTGACTCAGCACTATGATGCCAAGCCAACTGGCCGGGATTACAGATACTGCGAAAGTATGATGAAGAAAAGAA
M_musculus4    ACGAAAAATTCCTACGTCAGCACTATGATGCCAAGCCAAAGGGCCGGGACGACAGATACTGTGAAAGTATGATGAAGGAAAGAA
M_musculus5    ACAAAAATTTCCTGAATCAACACTATGATGCCAAGCCAACTGGCCGGGATTACAGATACTGTGAAAGTATGATGAAGAAAAGAA
M_musculus6    ACATAAAATTCCTGACTCAGCACTATGATGCCAAGCCAACAGGCTGGGATCACAGATACTGTTATCTGTGTATGATGAAGAAAA
R_norvegicus1  ACACGAAGTTCCTGACTCAGCACTATGATGCCAAGCCCAAGGGTCGGGATGCCAGATACTGCGAAAGTATGATGAGGAGAAGAG
R_norvegicus2  ACACGAAGTTCCTGACTCAGCACTATGATGCCAAGCCCAAGGGTCGGGATGCCAGATACTGCGAAAGCATGATGAAGAGAAGAG
T_francoisi    ACAGAGACTTCCTGACCAAGCACTATGATGCCACACCACAGGGCCGGAATGACAGATACTGTGAAAGCATGATGAGGAGACGGG
P_avunculus    ACAGAGACTTCCTGACCAAGCACTATGATGCCACACCACAGGGCCGGAATGACAGATACTGTGAAAGCATGATGAGGAGACGGG
P_bieti        ACAGAGACTTCCTGACCAAGCACTATGATGCCACACCACAGGGCCGGAATGACAGATACTGTGAAAGCATGATGAGGAGACGGG
P_roxellana    ACAGAGACTTCCTGACCAAGCACTATGATGCCACACCACAGGGCCGGAATGACAGATACTGTGAAAGCATGATGAGGAGACGGG
P_pygmaeus     ACACAGACTTCCTGGCCCAGCACTATGATCCCAAACCACAGGGCCGGGATGACAGATACTGTGAAAGCATCATGAGGAGACGGG
C_aethiops     ACAGAGACTTCCTGGCCAAGCACTATGATGCCACACCACAGGGCCGGAATGACAGATACTGTGAAAGCACGATGAGGAGACGAC
S_scrofa       ACACACACTTCCTGACCCAGCACTACGATGCCAAACCAAAGGGCCGGGATGGCAGATACTGTGAAAGCATAATGAAGCAACGAG
M_talapoin     ACAGAGACTTCCTGAGCAAGCACTATGATGCCAGACCACAGGGCCGGAATGACAGATACTGCGACAGCATGATGAGGAGACAGG
P_troglodytes  ACACACACTTCCTGACCCAGCACTATGATGCCAAACCACAGGGCCGGGATCACAGATACTGTGAAAGCATCATGAGGAGACGGG
M_mulata       ACAGAGACTTCCTGGCCAAGCACTATGATGCCACACCACAGGGCCGGAATGACAGATACTGTGAAAGCACAATGAGGAGACGGC
E_caballus     ACAGACAGTTCCTGACCAAGCACTATGATGCCAATCCAAGGGGCCGGAATGACAGATACTGTGAAAGCATGATGGTGAGACGAC
S_oedipus      ACAACAGGTTCCTGGAAGAGCACTATGATCCCAAAACAAAGAACGGGAATGACAGATACTGTGATAAAATGATGCGCTTAAGGA
B_taurus       ACATACACTTCCTGACCCAGCACTACGATGCCAAACCAAAGGGCCGGAATGACGAATATTGTTTTAACATGATGAAAAATCGAC
S_sciureus     ACATCAAGTTCCTGGACCAGCACTATGATCCCAAAACAAAGAACGGGAATGACAAATACTGTGAAAAAATGATGAGGTTAAGGA
A_trivirgatus  ACATCAAGTTCCTGGACCAGCACTATGATCCCAAAACAAAGAACGGGAATGACAAATACTGTGAAAAAATGATGAGGTTAAGGA
P_hamadryas    ACAGAGACTTCCTGGCCAAGCACTATGATGGCACACCAGAGGGCCGGAATGACAGATACTGTGAAAGCACGATGAGGAGACGGC
C_guereza      ACAGAGACTTCCTGACCAAGCACTATGATGCCACACCACAGGGCCGGAATGACAGATACTGTGAAAGCAAGATGAGGAAACTGG
M_domestica    AGAAACACTTCTTGATTCAGCACTATGACTCCAAACCAAAGGGCCGGGATGACAAGTACTGTACAGCGATCATGAGACAGAGAG

                    210       220       230       240       250       260       270       280
           ....|....|....|....|....|....|....|....|....|....|....|....|....|....|....|....|....
H_sapiens      ACCCTGCAAAGACATCAACACATTTATTCATGGCAACAAGCGCAGCATCAAGGCCATCTGTGAAAACAAGAATGGAAACCCTCA
M_musculus1    ACCCTGCAAAGATGTCAACACCTTTATCCATGGCAACAAGAGCAACATCAAGGCCATCTGTGGAGCGAAT---GGAAGCCCTTA
M_musculus2    TTTCTGCAAAGATGTCAACACCTTTATCCATGACACCAAGAACAACATCAAGGCCATCTGTGGAAAGAAA---GGAAGCCCTTA
M_musculus3    GCCTTGCAAAGAAGTCAACACCTTTATTCATGACACCAAGAACAACATCAAGGCCATCTGTGGAGAGAAT---GGAAGGCCTTA
M_musculus4    GCCTTGCAAAGATGTCAACACCTTTATCCATGGCACCAAGAAAAACATCAGGGCCATCTGTGGAAAGAAA---GGAAGCCCTTA
M_musculus5    ACCTTGCAAAGAAGTCAACACCTTTATCCATGACACCAAGAACAACATCAAGGCCATCTGTGGAGAGAAT---GGAAGGCCTTA
M_musculus6    GAAATGCAAAGAAGCCAACACCTTTATCCATGACACCAAGAAAAACATCAAGGCCATCTGTGGAGAGAAT---GGAGGCCCTTA
R_norvegicus1  GCCCTGCAAAGAGGTCAACACCTTTATCCATGGCAACAAGGGCAGCATCAAGGCCATCTGTGGCGCGAAT---GGAAGCCCTTA
R_norvegicus2  GCCCTGCAAAGAGGTCAACACCTTTATCCATGGCAATAAGGGCAGCATCAAGGCCGTCTGTGGTGCGAAT---GGAAGCCCTTA
T_francoisi    ACCCTGCAAAGACATCAACACCTTTATTCATGGCAACAGTCGCCACATCAAGGCCATCTGTGGAGATGAGAATGGAAACCCTTA
P_avunculus    ACCCTGCAAAGACATCAACACCTTTATTCATGGCAACAGTCGCCACATCAAGGCCATCTGTGGAGATGAGAATGGAAACCCTTA
P_bieti        ACCCTGCAAAGACATCAACACCTTTATTCATGGCAACAGTCGCCACATCAAGGCCATCTGTGGAGATGAGAATGGAAACCCTTA
P_roxellana    ACCCTGCAAAGACATCAACACCTTTATTCATGGCAACAGTCGCCACATCAAGGCCATCTGTGGAGATGAGAATGGAAACCCTTA
```

```
P_pygmaeus       ACCCTGCAAAGGCATCAACACATTTATTCATGGCAGCAAGCGCAGCATCAAGGCCATCTGTGAAAACAAGAATGGAAACCCTCA
C_aethiops       ACCCTGCAAAGACATCAACACCTTTATTCATGGCAACAGGCACCACATCAAGGCCATCTGTGGAGATGAGAATGGAAACCCTTA
S_scrofa         ACCCTGCAAAGAGGTCAACACCTTTATTCATGGCACGAGGAATGATATCAAGGCCATCTGTAATGATAAGAATGGAGAGCCTTA
M_talapoin       ACCCTGCAAAGACATCAACACCTTTATTCATGGCAACAGGCGCAGCATCAGGGCCATCTGTGGAGATGAGAATGGAAACCCTTA
P_troglodytes    ACCCTGCAAAGACATCAACACATTTATTCATGGCAACAAGCGCAGCATCAAGGCCATCTGTGAAAACAAGAATGGAAACCCTCA
M_mulata         ACCCTGCAAAGACATCAACACCTTTGTTCATGGCAACAGGCACCACATCACGGCCATCTGTGGAGATGAGAATGGAAGCCCTTA
E_caballus       ACCCTGCAAAGACACCAACACTTTTATTCATGGCAGCAAGAGCAGCATCAAGGCCATCTGTGGAAATAAGAATGGAAACCCTTA
S_oedipus        ACCCTGCAAAGCAATCAACACCTTTATTCATGGCAAGAAGGAGAGCATCAAGGCCATCTGTGGAACTGAGAATGGAGTGCCTTA
B_taurus         ACCTTGCAAAGACCGCAACACCTTTATTCATGGCAACAAGAATGACATTAAGGCCATCTGTGAGGACAGAAATGGACAGCCTTA
S_sciureus       ACCCTGCAAAGAAATCAACACCTTTATTCATGGCAACAAGGCCAGCATCAAGGCCATCTGTGGAAATCAGAATGGACAGCCTTA
A_trivirgatus    ACCCTGCAAAGAAATCAACACCTTTATTCATGGCAACAAGGCCAGCATCAAGGCCATCTGTGGAAATCAGAATGGAGAGCCTCA
P_hamadryas      ACCCTGCAAAGACACCAACACCTTTATTCATGGCAACAGGCACCACATCAACGCCATCTGTGGAGATGAGAATGGAAACCCTTA
C_guereza        ACCCTGCAAAGCCATCAACACCTTTATTCATGGCAACAGTCGCCACATCAAGGCCATCTGTAGAGATGAGAATGGAAACCCTTA
M_domestica      GCCCTGCAAGGATATGAATACCTTCATCCATGGTGATTACCCTAGCATCAAGGCTGTCTGTGGAGATAAAGCTGGGAATCCCTA

                          310       320       330       340       350       360       370       380
                 ....|....|....|....|....|....|....|....|....|....|....|....|....|....|....|....|....
H_sapiens        AGAATAAGCAAGTCTTCTTTCCAGGTCACCACTTGCAAGCTACATGGAGGTTCCCCCTGGCCTCCATGCCAGTACCGAGCCACAC
M_musculus1      AGAATGAGCAAGTCTCCCTTCCAGGTCACCACTTGCAAGCACACAGGAGGGTCTCCCCGGCCTCCATGCCAGTACCGAGCCTCTC
M_musculus2      AGAATAAGCAAGTCTCACTTCCAGGTCACCACTTGCACACACAAAGGAAGGTCTCCCCGGCCTCCATGCAGGTACCGAGCCTCTA
M_musculus3      AGAATAAGCAATTCTCGATTCCAGGTCACCACTTGCACGCACAAAGGAGGGTCTCCCAGGCCTCCATGCCAGTACAATGCCTTTA
M_musculus4      AGAATAAGCAATTCTCCCTTCCAGATCACCACTTGTACGCACTCAAGAGGGTCTCCCTGGCCTCCATGCGGGTACCGAGCCTTTA
M_musculus5      AGAATAAGCAATTCTCGCTTCCAGATCACAACTTGCAAGCACAAAGGAGGGTCTCCCAAGCCTCCATGCCAGTACAAAGCCTTTA
M_musculus6      AGAATAAGCAATTCTCCCTTCCAGATCACCACTTGCAATCACTCAGGAGGGTCTCCCAAGCCTCCATGCCAGTACAGAGACTTTA
R_norvegicus1    AGAATAAGCCAGTCTCCCTTCCAGATCACCACCTGCAAGCATACAGGAGGGTCTCCCCGGCCCCCTTGCCGGTACCGAGCCTCTC
R_norvegicus2    AGAATAAGCCAGTCTCCCTTCCAGATCACCACTTGCAAGCATACAGGAGGGTCTCCCCGGCCCCCTTGCCGGTACCGAGCCTCTC
T_francoisi      AGAATAAGCAAGTCTCCTTTCCAGGTCACCACTTGCAACCTACGTGGAGGATCCCCCTGGCCTCCATGCCGGTACCGAGCCACAC
P_avunculus      AGAATAAGCAAGTCTCCTTTCCAGGTCACCACTTGCAACCTACGTGGAGGATCCTCCCGGCCTCCATGCCGGTACCGAGCCACAC
P_bieti          AGAATAAGCAAGTCTCCTTTCCAGGTCACCACTTGCAACCTACGTGGAGGATCCTCCCGGCCTCCATGCCGGTACCGAGCCACAC
P_roxellana      AGAATAAGCAAGTCTCCTTTCCAGGTCACCACTTGCAACCTACGTGGAGGATCCTCCCGGCCTCCATGCCGGTACCGAGCCACAC
P_pygmaeus       AGAATAAGCAAGTCTTCTTTCCAGGTCACCACTTGCAAGCTACATGGAGGGTCCCCCTGGCCTCCATGCCACTACCGAGCCACAC
C_aethiops       AGAATAAGCAAGTCTCCTTTCCAGGTCACCACTTGCAACCTACGTGGAGGATCCCCCCGGCCTCCATGCCAGTACCGAGCCACAC
S_scrofa         AGAAGAAGCAAGTCTCCTTTCCAAATTACCACTTGCAAGCATAAGGGAGGGTCCAACCGGCCTCCATGTGGGTACAGGGCCACAC
M_talapoin       AGAATAAGCAGGACTCCTTTCCAGGTCACCACTTGCAACCTACGTGGAGGATCCCCCCGGCCTCCATGCCGGTACCGAGCCACAC
P_troglodytes    AGAATAAGCAAGTCTTCTTTCCAGGTCACCACTTGCAAGCTACATGGAGGGTCCCCCTGGCCTCCATGCCAGTACCGAGCCACAC
M_mulata         AGAATAAGCACGTCTCCTTTCCAGGTCACCACTTGCAAACTACGTGGAGGATCCCCCCGGCCTCCATGCCAGTACCGAGCCACAC
E_caballus       AGAATAAGCAAGACTCGTTTCCAGGTCACCACTTGCAAGCATGCAGGAGGGTCCCCCCGGCCTCCATGCCGATACAGAGCCACAC
S_oedipus        AGAAAAAGCAAGTCTGCTTTCCAGGTCACCATTTGCAAGCATAGAGGAGGGTCCCCCCGGCCTCCATGCCAGTACCGAGCCACAC
B_taurus         AGAATAAGCAAGTCTGAATTCCAGATCACCATCTGCAAGCATAAAGGAGGTTCCTCCCGGCCTCCATGCCGGTACGGAGCCACAC
S_sciureus       AGAATAAGCACGTCTGCTTTCCAGGTCACCATTTGCAGGCATATAGGAGGGTCCCCCCGGCCTCCATGCCGGTACCGAGCCACAC
A_trivirgatus    AGAATAAGCAAGTCTGCTTTCCAGGTCACCATTTGCAGGCATATAGGAGGGTCCCCCCGGCCTCCATGCCGGTACCGAGCCACAC
P_hamadryas      AGAATAAGCAAGTCTCCTTTTCAGGTCACCACTTGCAAGCTACATGGAGGATCCCCCCGGCCTCCATGCCGGTACCGAGCCACAC
C_guereza        AGAATAAGCAAGTCTCCTTTCCAGGTCACCACTTGCAACCTACGTGGAGGATCCCCCCGGCCTCCATGCCGGTACCGAGCCACAC
M_domestica      AGAATAAGCAAGTCACGATTTCAGGTCACTAACTGTGAGCATCGAGGAGGGTCTACAAAACCTCCCTGCAAGTACAGGGCCACTT

                          410       420       430       440       450       460       470       480
                 ....|....|....|....|....|....|....|....|....|....|....|....|....|....|....|....|....
H_sapiens        TTGTTGTTGCTTGTGAAAATGGCTTACCTGTCCACTTGGATCAGTCAATTTTCCGT------------------------------
M_musculus1      TTGTTATTGCCTGTGAGAATGGCTTGCCGGTCCACTTCGATGAGTCATTTTTC---------------------------------
M_musculus2      TTATTATTGGCTGTGAGAATGGCTGGCCTGTCCACTTTGATGAGTCTTTTATC---------------------------------
M_musculus3      TTGTTATTGCCTGTGAAGATGGCTGGCCTGTCCACTTCGATGAGTCTTTTATC---------------------------------
M_musculus4      TTGTTATTGCCTGTGAAGATGGCTGGCCTGTCCACTTCGATGAGTCTTTTATC---------------------------------
M_musculus5      TTGTTATTGCCTGTGAAGATGGCTGGCCTGTCCACTTCGATGAGTCTTTTATC---------------------------------
M_musculus6      TTGTTATTGCCTGTGAAGATGCCTGGCCTGTCCACTTCGATGAGTCTTTTATC---------------------------------
R_norvegicus1    TTGTTATTGCCTGTGAAAATGGCTTGCCTGTCCACTTTGATGAGTCTTTTATC---------------------------------
R_norvegicus2    TTGTTATTGCCTGTGAGAATGGCTTGCCTGTCCACTTTGATGAGTCTTTTATCAGTCTCTACTCAGCAGGCCCCCGGCGCAGAC
T_francoisi      TTGTTGTTGCTTGTGAAAATGACCTGCCTGTCCACTTGGATCAGTCAATTTTC---------------------------------
P_avunculus      TTGTTGTTGCTTGTGAAAATGACCTGCCTGTCCACTTGGATCAGTCAATTTTC---------------------------------
P_bieti          TTGTTGTTGCTTGTGAAAATGACCTGCCTGTCCACTTGGATCAGTCAATTTTC---------------------------------
P_roxellana      TTGTTGTTGCTTGTGAAAATGACCTGCCTGTCCACTTGGATCAGTCAATTTTC---------------------------------
P_pygmaeus       TTGTTGTTGCTTGTGAAAATGGCTTACCTGTCCACTTGGATCAGTCAATTTTCCGT------------------------------
C_aethiops       TTGTTGTTGGTTGTGAAAATGGCCTACCTGTCCACTTGGATGAGTCAATTTTC---------------------------------
S_scrofa         TAGCTGTTGCCTGTGAAAATGGCTTGCCTGTCCACTTTGATGAGTCCTTTATC---------------------------------
M_talapoin       TTGTTGTTGCTTGTGAAAATGGCCTACCTGTCCACTTGGATCAGTCAATTTTC---------------------------------
P_troglodytes    TTGTTGTTGCTTGTGAAAATGGCTTACCTGTCCACTTGGATCAGTCAATTTTCCGT------------------------------
M_mulata         TTGTTGTTGGTTGTGAAAATGGCCTACCTGTCCACTTGGATGAGTCCATTTTC---------------------------------
E_caballus       TTGTCATTGCCTGTGAAAACGGCTTGCCTGTCCACTTTGATGAGTCCTTTTTC---------------------------------
S_oedipus        TTGTAGTTGCCTGTGAAAATGGCTTACCTGTCCACTTAGATGAGTCCATTTTC---------------------------------
B_taurus         TTGTTGTCGGCTGTGAAAATGGCTTGCCCGTCCACTTTGATGAGTCCTTTATC---------------------------------
S_sciureus       TTGTTATTGCCTGTGAAAATGGCTTACCTGTCCACTTAGATGAGTCCATTTTC---------------------------------
A_trivirgatus    TTGTTGTTGCCTGTGAAAATGACTTACCTGTCCACTTAGATGAGTCCATTTTC---------------------------------
P_hamadryas      TTGTTGTTGGTTGTGAAAATGGCCTACCTGTCCACTTGGATGAGTCCATTTTC---------------------------------
C_guereza        TTGTTGTTGCTTGTGAAAATGACCTACCTGTCCACTTGGATCAGTCAATTTTC---------------------------------
M_domestica      TCATCATTGCCTGTGAGAATAATTTACCTGTCCACCTTGACCAGACCATCATT---------------------------------
```

```
                        510       520
               ....|....|....|....|....|
H_sapiens      -------------CGTCCG------
M_musculus1    -------------AGTCTA------
M_musculus2    -------------AGTCCA------
M_musculus3    -------------AGTCCG------
M_musculus4    -------------AGTCCG------
M_musculus5    -------------AGTATG------
M_musculus6    -------------AGTCTG------
R_norvegicus1  -------------AGTCTC------
R_norvegicus2  TTTATCTCCCCTCACCCCAGAACAC
T_francoisi    -------------CATCCG------
P_avunculus    -------------CGTCCG------
P_bieti        -------------CGTCCG------
P_roxellana    -------------CGTCCG------
P_pygmaeus     -------------CGTCTG------
C_aethiops     -------------CGTCCG------
S_scrofa       -------------ATTACAAGCCAG
M_talapoin     -------------CGTCCG------
P_troglodytes  -------------CGTCCG------
M_mulata       -------------CGTCCG------
E_caballus     -------------CGTCCA------
S_oedipus      -------------CGTCCA------
B_taurus       -------------ACTCCACGCCAC
S_sciureus     -------------CGTCCG------
A_trivirgatus  -------------CGTCCG------
P_hamadryas    -------------CGTCCG------
C_guereza      -------------CGTCCG------
M_domestica    -------------GCAAAA------
```
