# Supplementary material for: Mutational dynamics of murine angiogenin duplicates
Source: BMC Evol Biol. 2010 Oct 15;10:310. doi: 10.1186/1471-2148-10-310 (PMC2964713; doi:10.1186/1471-2148-10-310)
Supplement: Additional file 2 — Figure S2. Multiple sequence alignment for nucleotides and proteins [file 1471-2148-10-310-S2.pdf]

```
                    10        20        30        40        50        60        70        80
           ....|....|....|....|....|....|....|....|....|....|....|....|....|....|....|....|....
H_sapiens      MVM---GLGVLLLVFVLGLGLTPPTL-AQDNSRYTHFLTQHYDAKPQGRDDRYCESIMRRRGL--TSPCKDINTFIHGNKRSIK
M_musculus1    MAI---SPGPLFLIFVLGLVVIPPTL-AQDDSRYTKFLTQHHDAKPKGRDDRYCERMMKRRSL--TSPCKDVNTFIHGNKSNIK
M_musculus2    MAM---SPGPLFLVFLLGLVVIPPTL-SQDDSRYTKFLTQHYDAKPKGRDDRYCESMMVKRKL--TSFCKDVNTFIHDTKNNIK
M_musculus3    MVM---SPGSLLLVFLLSLDVIPPTL-AQDNYRYIKFLTQHYDAKPTGRDYRYCESMMKKRKL--TSPCKEVNTFIHDTKNNIK
M_musculus4    MTM---SPCPLLLVFVLGLVVIPPTL-AQNE-RYEKFLRQHYDAKPKGRDDRYCESMMKERKL--TSPCKDVNTFIHGTKKNIR
M_musculus5    MVI---SPGSLLLVFLLSLDVIPPTL-AQDNYRYKNFLNQHYDAKPTGRDYRYCESMMKKRKL--TSPCKEVNTFIHDTKNNIK
M_musculus6    MVM---SPGSLMLVFVLSLVVISPTL-AKDNYRYIKFLTQHYDAKPTGWDHRYCYLCMMKKRIQETLKCKEANTFIHDTKKNIK
R_norvegicus1  MEM---SLRPLLSVFVLGLVSTPSTL-AQDDPRYTKFLTQHYDAKPKGRDARYCESMMRRRGL--TSPCKEVNTFIHGNKGSIK
R_norvegicus2  MEM---SLRPLLLVFVLSLVATPSTL-AQDDPRYTKFLTQHYDAKPKGRDARYCESMMKRRGL--TSPCKEVNTFIHGNKGSIK
T_francoisi    MVM---GLGLFLLVFMLGLGLTPPTL-AQDNSRYRDFLTKHYDATPQGRNDRYCESMMRRRGL--TSPCKDINTFIHGNSRHIK
P_avunculus    MVM---GLGLFLLVFMLGLGLTPPTL-AQDNSRYRDFLTKHYDATPQGRNDRYCESMMRRRGL--TSPCKDINTFIHGNSRHIK
P_bieti        MVM---GLGLFLLVFMLGLGLTPPTL-AQDNSRYRDFLTKHYDATPQGRNDRYCESMMRRRGL--TSPCKDINTFIHGNSRHIK
P_roxellana    MVM---GLGLFLLVFMLGLGLTPPTL-AQDNSRYRDFLTKHYDATPQGRNDRYCESMMRRRGL--TSPCKDINTFIHGNSRHIK
P_pygmaeus     MVM---GLGVLLLVFMLGLGLTPPTL-AQDNSRYTDFLAQHYDPKPQGRDDRYCESIMRRRGL--TSPCKGINTFIHGSKRSIK
C_aethiops     MVM---GLGLFLLVFMLGLGLTPPTL-AQDNSRYRDFLAKHYDATPQGRNDRYCESTMRRRHL--TSPCKDINTFIHGNRHHIK
S_scrofa       MVI---LLGPLLLVFMLGLGLAPLSL-AKDEDRYTHFLTQHYDAKPKGRDGRYCESIMKQRGL--TRPCKEVNTFIHGTRNDIK
M_talapoin     MVM---GLGLFLLVFMLGLGLTSPTL-AQDNSRYRDFLSKHYDARPQGRNDRYCDSMMRRQGM--TSPCKDINTFIHGNRRSIR
P_troglodytes  MVM---GLGVLLLVFVLGLGLTPPTL-AQDNSRYTHFLTQHYDAKPQGRDHRYCESIMRRRGL--TSPCKDINTFIHGNKRSIK
M_mulata       MVM---GLGLFLLVFMLGLGLTPPTL-AQDNPRYRDFLAKHYDATPQGRNDRYCESTMRRRHL--TSPCKDINTFVHGNRHHIT
E_caballus     MAM---SLCPLLLVFVLGLGLTPPSL-AQDDSRYRQFLTKHYDANPRGRNDRYCESMMVRRHL--TTPCKDTNTFIHGSKSSIK
S_oedipus      MVM---GLHLLFLVFILGLGLTPPTL-AQNDLRYNRFLEEHYDPKTKNGNDRYCDKMMRLRNM--ISPCKAINTFIHGKKESIK
B_taurus       MVM---VLSPLFLVFILGLGLTPVAP-AQDDYRYIHFLTQHYDAKPKGRNDEYCFNMMKNRRL--TRPCKDRNTFIHGNKNDIK
S_sciureus     MVM---GPHLLLLVFILGLGLTPPTL-AQNDSRYIKFLDQHYDPKTKNGNDKYCEKMMRLRNM--ISPCKEINTFIHGNKASIK
A_trivirgatus  MVM---GLHLLLLVFILGLGLTPPTL-AQNDIRYIKFLDQHYDPKTKNGNDKYCEKMMRLRNM--ISPCKEINTFIHGNKASIK
P_hamadryas    MVM---GLGLFLLVFMLGLGLTLPTL-AQDNFRYRDFLAKHYDGTPEGRNDRYCESTMRRRHL--TSPCKDTNTFIHGNRHHIN
C_guereza      MVM---GLGLFLLVFLLGLGLTPPTL-ALDNPRYRDFLTKHYDATPQGRNDRYCESKMRKLGL--TSPCKAINTFIHGNSRHIK
M_domestica    MVMFLKAMDPFLLLFILGLWLVPPSLTTQANAREKHFLIQHYDSKPKGRDDKYCTAIMRQRGL--TQPCKDMNTFIHGDYPSIK

                   110       120       130       140       150       160       170
           ....|....|....|....|....|....|....|....|....|....|....|....|....|....|....|
H_sapiens      RISKSSFQVTTCKLHGGSPWPPCQYRATAGFRNVVVACENGLPVHLDQSIFR-------------------RP--
M_musculus1    RMSKSPFQVTTCKHTGGSPRPPCQYRASAGFRHVVIACENGLPVHFDESFF--------------------SL--
M_musculus2    RISKSHFQVTTCTHKGRSPRPPCRYRASKGFRYIIIGCENGWPVHFDESFI--------------------SP--
M_musculus3    RISNSRFQVTTCTHKGGSPRPPCQYNAFKDFRYIVIACEDGWPVHFDESFI--------------------SP--
M_musculus4    RISNSPFQITTCTHSRGSPWPPCGYRAFKDFRYIVIACEDGWPVHFDESFI--------------------SP--
M_musculus5    RISNSRFQITTCKHKGGSPKPPCQYKAFKDFRYIVIACEDGWPVHFDESFI--------------------SM--
M_musculus6    RISNSPFQITTCNHSGGSPKPPCQYRDFKDFRYIVIACEDAWPVHFDESFI--------------------SL--
R_norvegicus1  RISQSPFQITTCKHTGGSPRPPCRYRASAGFRHVVIACENGLPVHFDESFI--------------------SL--
R_norvegicus2  RISQSPFQITTCKHTGGSPRPPCRYRASAGFRHVVIACENGLPVHFDESFISLYSAGPRRRPSSVLFLSPLTPEH
T_francoisi    RISKSPFQVTTCNLRGGSPWPPCRYRATAGFRNIVVACENDLPVHLDQSIF--------------------HP--
P_avunculus    RISKSPFQVTTCNLRGGSSRPPCRYRATAGFRNIVVACENDLPVHLDQSIF--------------------RP--
P_bieti        RISKSPFQVTTCNLRGGSSRPPCRYRATAGFRNIVVACENDLPVHLDQSIF--------------------RP--
P_roxellana    RISKSPFQVTTCNLRGGSSRPPCRYRATAGFRNIVVACENDLPVHLDQSIF--------------------RP--
P_pygmaeus     RISKSSFQVTTCKLHGGSPWPPCHYRATADFRNIVVACENGLPVHLDQSIFR-------------------RL--
C_aethiops     RISKSPFQVTTCNLRGGSPRPPCQYRATRGSRNIVVGCENGLPVHLDESIF--------------------RP--
S_scrofa       RRSKSPFQITTCKHKGGSNRPPCGYRATAGFRTIAVACENGLPVHFDESFI--------------------ITSQ
M_talapoin     RISRTPFQVTTCNLRGGSPRPPCRYRATAGFRNIVVACENGLPVHLDQSIF--------------------RP--
P_troglodytes  RISKSSFQVTTCKLHGGSPWPPCQYRATAGFRNVVVACENGLPVHLDQSIFR-------------------RP--
M_mulata       RISTSPFQVTTCKLRGGSPRPPCQYRATRGSRNIVVGCENGLPVHLDESIF--------------------RP--
E_caballus     RISKTRFQVTTCKHAGGSPRPPCRYRATPGFRSIVIACENGLPVHFDESFF--------------------RP--
S_oedipus      RKSKSAFQVTICKHRGGSPRPPCQYRATAGSRNVVVACENGLPVHLDESIF--------------------RP--
B_taurus       RISKSEFQITICKHKGGSSRPPCRYGATEDSRVIVVGCENGLPVHFDESFI--------------------TPRH
S_sciureus     RISTSAFQVTICRHIGGSPRPPCRYRATAGFRNIVIACENGLPVHLDESIF--------------------RP--
A_trivirgatus  RISKSAFQVTICRHIGGSPRPPCRYRATAGFRNLVVACENDLPVHLDESIF--------------------RP--
P_hamadryas    RISKSPFQVTTCKLHGGSPRPPCRYRATRGSRNIVVGCENGLPVHLDESIF--------------------RP--
C_guereza      RISKSPFQVTTCNLRGGSPRPPCRYRATAGFRNIVVACENDLPVHLDQSIF--------------------RP--
M_domestica    RISKSRFQVTNCEHRGGSTKPPCKYRATSDFRYIIIACENNLPVHLDQTII--------------------AK--
```
